# Assessment of bidirectional relationships between 98 genera of the human gut microbiota and amyotrophic lateral sclerosis: a 2-sample Mendelian randomization study

**DOI:** 10.1186/s12883-021-02522-z

**Published:** 2022-01-03

**Authors:** Linjing Zhang, Zhenhuang Zhuang, Gan Zhang, Tao Huang, Dongsheng Fan

**Affiliations:** 1 Department of Neurology, Peking University Third Hospital, 49 North Garden Road, Haidian District, Beijing, 100191 China; 2 Beijing Municipal Key Laboratory of Biomarker and Translational Research in Neurodegenerative Diseases, Beijing, China; 3 Department of Epidemiology & Biostatistics, School of Public Health, Peking University, Beijing, 100191 China; 4 Key Laboratory of Molecular Cardiovascular Sciences (Peking University), Ministry of Education, Beijing, China

**Keywords:** Amyotrophic lateral sclerosis, Gut microbiota, Gamma-glutamyl amino acids, Bidirectional relationships, Two-sample Mendelian randomization

## Abstract

**Background:**

Growing evidence suggests a mutual interaction between gut microbiome alterations and ALS pathogenesis. However, previous studies were susceptible to potential confounding factors and reverse causation bias, likely leading to inconsistent and biased results.

**Objectives:**

To decipher the potentially mutual relationship between gut microbiota and ALS, we used a bidirectional two-sample MR approach to examine the associations between the gut microbiome and ALS.

**Results:**

Using the inverse variance-weighted method, *OTU10032 unclassified Enterobacteriaceae species-level* OTU and *unclassified Acidaminococcaceae* were associated with a higher risk of ALS (per relative abundance: OR, 1.04; 95% CI, 1.01–1.07; *P* = 0.011 and OR, 1.02; 95% CI, 1.01–1.04; *P* = 0.009, respectively). Importantly, Gamma-Glu-Phe was showed potential deleterious effects on the risk of ALS (genetically predicted per a 1-standard deviation increase in the level of Gamma-Glu-Phe: OR, 1.96; 95% CI, 1.50–2.55; *P* = 0.012). Sensitivity analysis of the two candidate genera and metabolites using the MR-Egger and weighted-median methods produced similar estimates, and no horizontal pleiotropy or outliers were observed. Intriguingly, genetically predicted ALS was associated with an increase in the relative abundance of *OTU4607_Sutterella* (per 1-unit higher log odds: β, 2.23; 95% CI, 1.27–3.18; *P* = 0.020) and *Lactobacillales_ORDER* (per 1-unit higher log odds: β, 0.51; 95% CI, 0.09–0.94; *P* = 0.019).

**Conclusions:**

Our findings provide novel evidence supporting the bidirectional relationship between the gut microbiota and ALS. These results may contribute to designing microbiome- and microbiome-dependent metabolite interventions in future ALS clinical trials.

**Supplementary Information:**

The online version contains supplementary material available at 10.1186/s12883-021-02522-z.

## Background

**Fig. 1:**
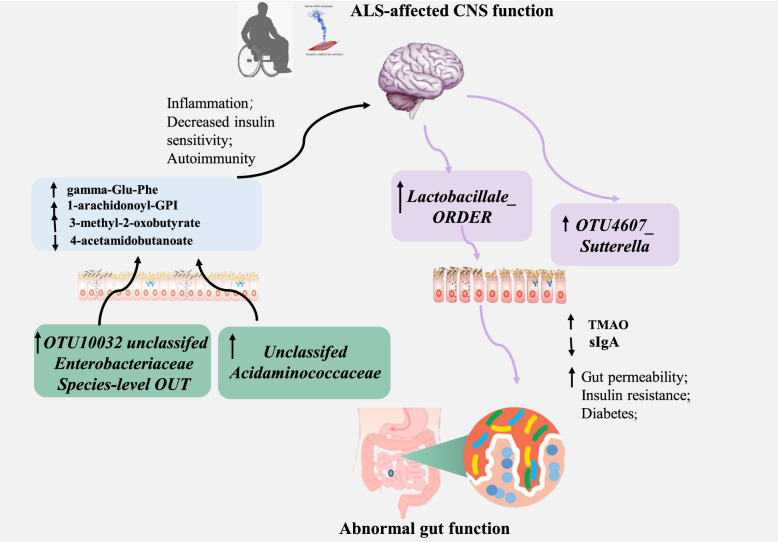
Graphic Abstract

Amyotrophic lateral sclerosis (ALS) is a fatal neurodegenerative motor neuron disease accompanied by both systemic and central nervous system–specific inflammation as well as energy dysmetabolism [1–3]. Structural components of the bacteria and various metabolites (pro-inflammatory cytokines or anti-inflammatory) secreted by the gut microbiota can stimulate or inhibit a cascade of inflammatory pathways on both a local and systemic scale [4]. Additionally, by-products of metabolic processes in bacteria, including some short-chain fatty acids, can play a role in inhibiting inflammatory processes [5]. These local and systemic inflammatory, which in turn could lead to perturbed gut-microbiota (dysbiosis) and increased intestinal permeability (leaky-gut) [6]. These potential pathogenetic factors have recently been found to mutually interact with the gut microbiota [7, 8], suggesting that the gut microbiota could be involved in the development of the disease and be affected by the disease vice versa (Fig. 1).

Observational studies have shown that the interface between the host and the gut microbiome may be altered in mouse models of ALS [9, 10], including impaired gut barrier function and a dysbiotic microbiome configuration that can be partially corrected by butyrate supplementation [10]. Studies of whether gut microbiome dysbiosis occurs between ALS patients and healthy controls have yielded conflicting results [11–13]. Notably, a recent study [14] of 11 distinct commensal bacteria based on their individual supplementation into antibiotic-treated *Sod1-Tg* mice found that *Akkermansia muciniphila* (AM) and AM-associated nicotinamide ameliorate symptoms of ALS. In humans, distinct microbiome and metabolite configurations have been observed in a small preliminary study that compared 37 patients with ALS with household controls [14].

Growing but conflicting evidence is attractive, raising the hypothesis of a mutual interaction between gut microbiome alterations and ALS pathogenesis. However, it has been difficult to determine whether these changes in the intestinal microbiota are causative of ALS disease, an exacerbating factor for disease, or a consequence of disease. The composition and diversity of the gut microbiome can be easily altered as a result of bacterial infections, antibiotic treatment, lifestyle changes, surgery, and long-term changes in diet [4]. Available evidence is in large part inadequate, as observational studies are susceptible to these potential confounding and reverse causation biases, which can lead to inconsistent and biased results [15–17]. To some extent, data from antibiotic-treated *Sod1-Tg* mice could demonstrate causal relationships but are scarce, and the number of commensal bacteria that have been investigated is limited [14].

The Mendelian randomization (MR) approach is a widely used genetic epidemiological method for assessing causal associations between risk factors and disease by exploiting genetic variants as instrumental variables (IVs) for exposure [18–20]. This approach is less likely to be affected by the confounding or reverse causation bias that exists in observational findings.

**Fig. 2:**
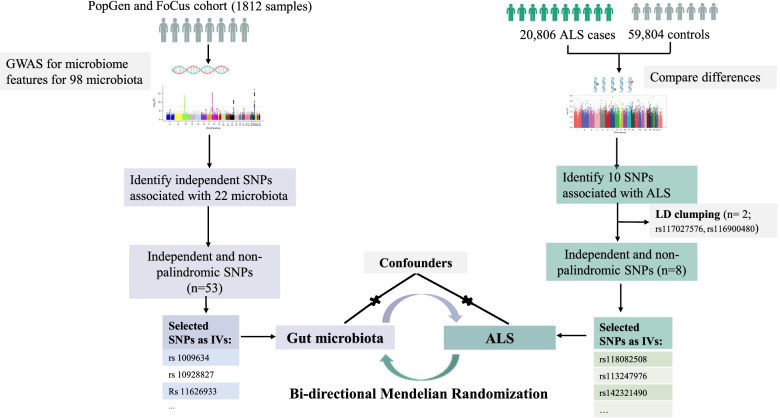
Schematic representation of the study

Therefore, to decipher the potentially mutual relationship between the gut microbiota and ALS, we used a bidirectional two-sample MR approach to examine the associations between the gut microbiome and ALS (Fig. 2**)**. Notably, the gut microbiome is remote from the disease site of ALS, it is suggested that a potential systemic influx of microbiome-regulated metabolites may affect the susceptibility of motor neurons in ALS. We also estimated the effects of potential metabolites on ALS in MR design.

## Methods

The detailed approach of selection of IVs for exposures, genome-wide association study (GWAS) summary statistics for ALS, and MR analysis were previously described [21]. The MR approach we used was based on the following three assumptions: 1) genetic variants (single nucleotide polymorphisms (SNPs)) used as IVs are associated with exposures; 2) genetic variants are not associated with confounders; and 3) genetic variants influence the risk of outcomes only through interested exposures, not through other pathways [22] (Fig. 2). The IVs (F statistic > 10) for all the exposures were sufficiently informative [23].

### Genetically predicted gut microbiota genera

Genetic instruments of the abundance of 98 genera of gut microbiota at the level of genome-wide significance (*P* < 5 × 10^− 8^) were obtained from available GWAS data of stool samples in humans [24]. As a result, independently significant SNPs were identified for 22 genera of the gut microbiota, but no significant genetic variants were found for the remaining 76 genera of the gut microbiota.

If an SNP was not available for an outcome, a highly correlated proxy SNP (r^2^ > 0.9) (https://ldlink.nci.nih.gov/) was used instead, if available. We checked the phenotypes of selected SNPs using comprehensive genotype-to-phenotype cross-references (GWAS Catalog [25]) and repeated the analysis with potentially pleiotropic SNPs excluded. We calculated SNP-specific *F* statistics as a quotient of squared SNP-genus association and its variance [26].

### Genetically predicted gut microbial metabolites

A transsynaptic, glutaminergic, excitotoxic mechanism (the so-called dying-forward hypothesis) has been proposed as a pathophysiological biomarker in ALS [27]. We therefore used 18 potential blood metabolites that might have causal effects on the development of ALS, including a group of gamma-glutamyl amino acids [28]. The candidate metabolites were identified among 486 untargeted serum metabolites from Shin’s study [29]. A total of 7824 adult individuals from 2 European cohorts were included in the GWAS analysis. Metabolomics data were acquired based on nontargeted mass spectrometry analysis of human fasting serum [29].

For each of the metabolites, we selected SNPs that showed an association at *P* < 1 × 10^− 5^ as candidate IVs of the specific metabolite. Then, a clumping procedure was conducted with European 1000G as a reference panel to identify the independent variants, with a linkage disequilibrium threshold of r^2^ < 0.01 in a 500-kb window.

### Genetically predicted ALS

We drew on summary statistics from the largest and most recent GWAS of ALS [30] patients who were defined as having been diagnosed with probable or definite ALS according to the El Escorial criteria (Brooks, 1994) by a neurologist specializing in ALS. This GWAS of ALS involving 20,806 patients and 59,804 controls of European ancestry identified 10 independent genome-wide significant SNPs at the level of *P* < 5 × 10^− 8^ [30].

### Statistical analysis

For each direction of the potential relationship, we combined MR estimates using an inverse variance-weighted method (IVW) meta-analysis, which essentially translates to a weighted regression of SNP outcome effects on SNP exposure effects where the intercept is constrained to zero. The IV assumptions can be biased if instrument SNPs show horizontal pleiotropy, influencing the outcome through causal pathways other than exposure [22]. Therefore, other established MR methods, including weighted, weighted median mode, and MR Egger regression, were also applied to confirm the IVW results (number of SNPs ≥3) because their estimates are known to be relatively robust to horizontal pleiotropy, although at the cost of reduced statistical power [31]. MR Egger regression allows the intercept to be freely estimated as an indicator of average pleiotropic bias. Effect estimates are reported in β values when the outcome is continuous (i.e., the abundance of each genus of gut microbiota) and are converted to ORs when the outcome is dichotomous (i.e., ALS status).

To assess the robustness of significant results, we conducted further tests for horizontal pleiotropy using meta-analytic methods to detect heterogeneous outcomes, including leave-1-SNP-out analyses and the MR Egger intercept test of deviation from the null [32].

The analyses were performed with R version 3.1.1 (R foundation) and Stata version 11.2 (Stata Corp, College Station, TX). All human research was approved by the relevant institutional review boards and conducted according to the Declaration of Helsinki. Ethical approval was obtained from relevant Research Ethics Committees and from the review boards of Peking University Third Hospital.

## Results

### Effects of genetically predicted gut microbiota on ALS

The resulting lists of instrument SNPs for each genus of gut microbiota are given in Table 1.

**Table 1:** Characteristics of selected SNPs for core gut microbiota

Core gut microbiota	SNP	Chr.	Locus start	Locus end	A1	A2	P	Beta	SE	β-div ***P***	Nearest gene	Genes in locus	Variance explained
OTU10032 unclassifed Enterobacteriaceae Species-level OTU	rs1009634	12	4,779,313	4,900,344	G	A	7.12E-09	−1.3	0.23	0.93	AKAP3	NDUFA9, GALNT8, RP11-234B24.2	0.0183
Bacilli class	rs10928827	2	129,426,740	129,473,850	G	A	1.02E-08	−0.2	0.04	0.19	HS6ST1	–	0.0180
Lactobacillales order	rs10928827	2	129,426,740	129,473,850	G	A	4.19E-09	−0.2	0.04	0.19	HS6ST2	–	0.0189
Unclassifed Erysipelotrichaceae	rs11626933	14	90,681,816	90,810,659	G	A	1.83E-08	−0.2	0.04	0.55	C14orf102	C14orf102	0.0173
Marinilabiliaceae family	rs11724031	4	77,441,448	77,467,405	G	A	2.44E−10	−1	0.15	0.68	SHROOM3	SHROOM3	0.0219
Unclassifed Marinilabiliaceae	rs11724031	4	77,441,448	77,467,405	G	A	2.44E-10	-1	0.15	0.68	SHROOM3	SHROOM3	0.0219
Erysipelotrichaceae family	rs11877825	18	10,566,345	10,595,758	G	T	2.82E-11	−0.3	0.04	0.34	NAPG	–	0.0242
Erysipelotrichia class	rs11877825	18	10,566,345	10,595,758	G	T	2.82E-11	−0.3	0.04	0.34	NAPG	–	0.0242
Erysipelotrichales order	rs11877825	18	10,566,345	10,595,758	G	T	2.82E-11	−0.3	0.04	0.34	NAPG	–	0.0242
Marinilabiliaceae family	rs11915634	3	1,452,602	1,517,331	T	C	2.99E-10	−1.3	0.21	0.14	CNTN6	–	0.0217
Unclassifed Marinilabiliaceae	rs11915634	3	1,452,602	1,517,331	T	C	2.99E-10	−1.3	0.21	0.14	CNTN7	–	0.0217
OTU10032 unclassifed Enterobacteriaceae	rs12149695	16	27,205,994	27,293,886	A	T	1.82E-09	0.61	0.10	0.23	FLJ21408	NSMCE1, FLJ21408, KDM8	0.0198
OTU15355 Dialister Species-level OTU	rs12442649	15	37,968,393	38,035,538	G	A	3.72E-08	−1.5	0.27	0.85	TMCO5A	–	0.0166
EscherichiaShigella	rs13096731	3	58,014,818	58,089,851	A	G	2.55E-08	−0.4	0.08	0.12	FLNB	FLNB	0.0170
OTU10032 unclassifed Enterobacteriaceae	rs13276516	8	56,589,428	56,596,140	A	G	5.54E-09	− 0.6	0.10	0.41	TGS1	–	0.0186
Lactobacillales order	rs1362404	16	51,955,443	52,017,380	T	G	1.56E-08	0.23	0.04	7.50E-05	TOX3	–	0.0175
Bacilli class	rs148330122	19	38,497,288	38,631,252	C	T	1.32E-09	−0.5	0.08	0.18	SIPA1L3	SIPA1L3	0.0201
OTU10032 unclassifed Enterobacteriaceae	rs17085775	9	71,165,704	71,167,878	C	T	2.06E-08	−1	0.18	0.54	C9orf71	–	0.0172
Erysipelotrichaceae family	rs17421787	4	131,293,675	131,512,291	C	G	3.60E-08	−0.3	0.05	0.16	RP11-22 J15.1	–	0.0166
Erysipelotrichales order	rs17421787	4	131,293,675	131,512,291	C	G	3.60E-08	−0.3	0.05	0.16	RP11-22 J15.2	–	0.0166
Erysipelotrichia class	rs17421787	4	131,293,675	131,512,291	C	G	3.60E-08	−0.3	0.05	0.16	RP11-22 J15.3	–	0.0166
Unclassifed Acidaminococcaceae	rs17661843	7	48,381,902	48,433,594	T	C	3.72E-14	−1.4	0.18	0.26	ABCA13	ABCA13	0.0312
Bacilli class	rs2071199	20	43,030,809	43,037,422	T	C	1.24E-08	−0.3	0.06	0.58	HNF4A–AS1	HNF4A	0.0178
OTU10032 unclassifed Enterobacteriaceae Species-level OTU	rs2318350	8	139,889,972	139,942,500	T	C	3.65E-09	−1.2	0.19	0.95	COL22A1	COL22A1	0.0190
OTU10032 unclassifed Enterobacteriaceae	rs249733	5	141,877,862	141,911,748	T	C	4.74E-10	−0.7	0.10	0.68	SPRY4	–	0.0212
Actinobacteria class	rs34613612	21	32,184,901	32,204,347	C	G	6.34E-10	0.25	0.04	9.87E-03	KRTAP8–1	KRTAP8–1	0.0209
Actinobacteria phylum	rs34613612	21	32,184,901	32,204,347	C	G	6.34E-10	0.25	0.04	9.87E-03	KRTAP8–1	KRTAP8–1	0.0209
Enterobacteriaceae family	rs35275482	15	60,027,987	60,128,040	C	A	3.72E-11	−0.5	0.08	0.06	BNIP2	–	0.0239
Enterobacteriales order	rs35275482	15	60,027,987	60,128,040	C	A	3.72E-11	−0.5	0.08	0.06	BNIP3	–	0.0239
OTU10032 unclassifed Enterobacteriaceae Species-level OTU	rs3925158	3	38,161,078	38,313,688	C	G	6.29E-09	−1	0.17	0.78	SLC22A13	SLC22A13, MYD88, DLEC1, ACAA1, OXSR1	0.0185
Gammaproteobacteria class	rs4621152	2	217,857,450	217,924,261	C	T	1.40E-08	−0.3	0.05	0.79	AC007557.1	–	0.0176
Blautia genus	rs4669413	2	9,801,744	9,818,596	T	C	1.20E-08	−0.2	0.03	0.75	RP11–521D12.1	–	0.0178
Bacilli class	rs479105	12	3,357,596	3,393,503	T	C	1.21E-08	−0.2	0.04	0.48	PRMT8	–	0.0178
Unclassifed Acidaminococcaceae	rs56006724	2	228,486,044	228,523,585	A	G	6.35E-10	−0.9	0.14	0.93	C2orf83	C2orf83	0.0209
Lactobacillales order	rs59042687	3	95,359,287	95,823,523	T	G	6.22E-09	−0.2	0.04	0.02	LINC00879	–	0.0185
OTU13305 Fecalibacterium Species-level OTU	rs597205	1	112,379,026	112,415,622	T	C	7.68E-09	−0.6	0.11	0.85	C1orf183	C1orf183	0.0183
Lactobacillales order	rs62295801	3	162,444,724	163,236,170	G	T	5.32E-10	−0.3	0.04	0.21	LINC01192	LINC01192	0.0211
Lactobacillales order	rs7083345	10	7,020,329	7,044,987	T	C	2.89E-09	0.24	0.04	0.02	RP11-554I8.2	–	0.0199
Bacilli class	rs7083345	10	7,020,329	7,044,987	T	C	3.38E-10	0.25	0.04	0.02	RP11-554I8.2	–	0.0209
Lactobacillales order	rs7113056	11	122,091,502	122,154,110	C	T	1.72E-13	−0.5	0.07	0.07	RP11-166D19.1	–	0.0296
Unclassifed Acidaminococcaceae	rs75036654	1	37,717,219	37,780,821	C	T	4.94E-10	−1.4	0.22	0.06	LINC01137	–	0.0212
Bacilli class	rs7646786	3	185,729,634	185,742,372	T	C	2.29E-08	−0.2	0.04	0.5	LOC344887	–	0.0171
Unclassifed Porphyromonadaceae	rs7656342	4	9,721,358	9,895,176	A	G	2.80E-09	0.39	0.07	0.22	DRD5	SLC2A9, DRD5	0.0193
Blautia genus	rs79387448	2	103,099,953	103,239,356	C	T	7.68E-11	−0.3	0.05	0.66	SLC9A2	SLC9A2	0.0231
Unclassifed Porphyromonadaceae	rs9291879	5	66,515,817	66,550,855	C	T	3.51E-09	−0.6	0.10	0.08	CD180	–	0.0191
Gammaproteobacteria class	rs9300430	13	98,269,478	98,306,405	C	T	1.30E-09	−0.6	0.10	0.12	RAP2A	–	0.0201
Proteobacteria phylum	rs9323326	14	58,476,448	58,532,709	A	G	8.76E-10	−0.2	0.03	0.02	SLC35F4	C14orf37	0.0206
Unclassifed Enterobacteriaceae	rs938295	1	16,087,164	16,124,985	C	T	2.34E-08	−0.5	0.09	0.76	FBLIM1	FBLIM1	0.0171
Unclassifed Marinilabiliaceae	rs9831278	3	98,879,786	98,942,990	C	T	2.53E-08	−1.2	0.21	0.49	LINC00973	–	0.0170
Marinilabiliaceae family	rs9831278	3	98,879,786	98,942,990	C	T	2.53E-08	−1.2	0.21	0.49	LINC00974	–	0.0170
Unclassifed Acidaminococcaceae	rs986417	14	60,787,269	61,122,040	C	T	2.63E-09	−1.4	0.23	0.47	SIX6	SIX6, C14orf39, SIX1	0.0194
Marinilabiliaceae family	rs9996716	4	77,441,448	77,467,405	G	A	5.58E-09	−0.7	0.12	0.2	SHROOM3	SHROOM3	0.0186
Unclassifed Marinila-biliaceae	rs9996716	4	77,441,448	77,467,405	G	A	5.58E-09	−0.7	0.12	0.2	SHROOM3	SHROOM3	0.0186

The 53 associations with bacterial abundance are grouped into 40 loci on the basis of LD. *SNP* single-nucleotide polymorphisms, *Chr* chromosome, *A1* effect allele, *A2* non-effect allele, *P* meta-analysis *P* value for A1, *Beta* meta-analysis coeffcient for A1, *SE* standard error, *β-div P* P value for association with β diversity.

**Fig. 3:**
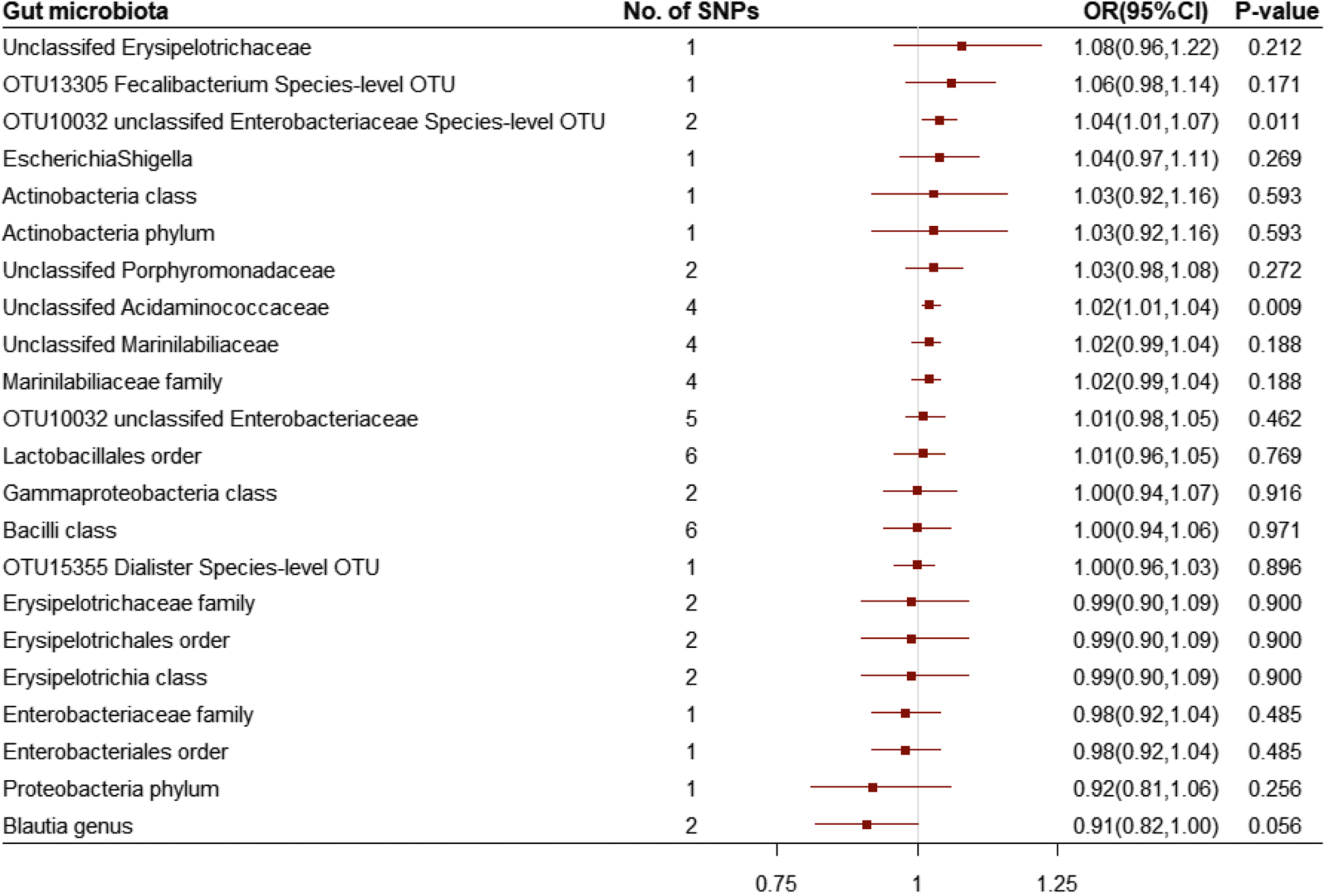
Odds ratio for association of genetically predicted gut microbiota with amyotrophic lateral sclerosis. OR: odds ratio; CI: confidence internal. OR (95% CI) means risk of amyotrophic lateral sclerosis per 1-allele increase in single nucleotide polymorphisms related to greater abundance of gut microbiota

On the basis of 2 independent SNPs, *OTU10032 unclassified Enterobacteriaceae* was associated with a higher risk of ALS (per relative abundance: OR, 1.04; 95% CI, 1.01–1.07; *P* = 0.011) (Fig. 3, eFigure 1). Additionally, on the basis of 4 uncorrelated SNPs, *unclassified Acidaminococcaceae* was associated with a higher risk of ALS (per relative abundance: OR, 1.02; 95% CI, 1.01–1.04; *P* = 0.009) (Fig. 3, eFigure 2). The independent SNPs for two genera with r ^2^ = 0 are listed in eTable 1. Sensitivity analysis for the two candidate genera using the MR-Egger and weighted-median methods produced similar estimates, and no horizontal pleiotropy or outliers were observed (eTable 2–3).

**Fig. 4:**
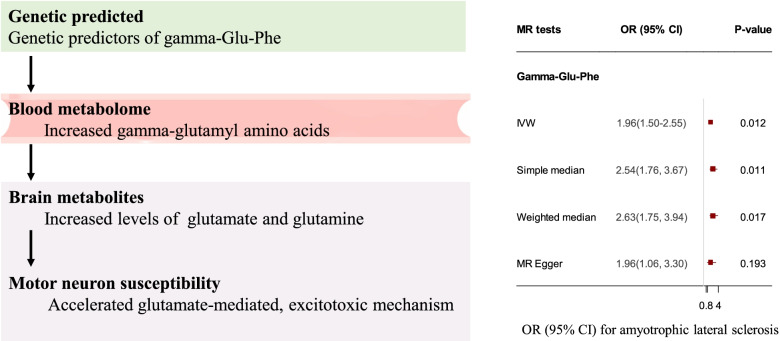
Causal effect of microbiome-dependent metabolites on the risk of ALS. OR: odds ratio; CI: confidence internal

Importantly, gamma-glutamyl amino acids showed potential deleterious effects on the risk of ALS. Gamma-glutamylphenylalanine (Gamma-Glu-Phe), a peptide in the gamma-glutamyl pathway, showed a significantly increased risk of ALS (genetically predicted per 1-standard deviation (SD) increase in the level of Gamma-Glu-Phe: OR, 1.96; 95% CI, 1.50–2.55; *P* = 0.012) (Fig. 4). In addition, two metabolites, 1-arachidonoyl-GPI and 3-methyl-2-oxobutyrate, were also estimated to be associated with a higher risk of ALS, with a genetically predicted per 1-SD increase in levels: OR, 1.64; 95% CI, 1.37–1.96; *P* = 0.005 for 1-arachidonoyl-GPI and OR, 2.78; 95% CI, 1.98–3.90; *P* = 0.003 for 3-methyl-2-oxobutyrate. The results also showed that a genetically predicted increase in the levels of 4-acetamidobutanoate may lower the risk of ALS (per 1-SD increase in levels: OR, 0.49; 95% CI, 0.36–0.66; *P* = 0.020). Sensitivity analysis for the metabolites using the MR-Egger and weighted-median methods produced similar estimates, and no horizontal pleiotropy or outliers were observed (eTable 4).

### Effects of genetically predicted ALS on gut microbiota

On the basis of 2 independent SNPs, genetically predicted ALS was associated with an increase in the relative abundance of *OTU4607_Sutterella* (per 1-unit higher log odds: β, 2.23; 95% CI, 1.27–3.18; *P* = 0.020). The risk of ALS on each *OTU4607_Sutterella*-related SNP effect was estimated and is shown in eFigure 3. Similarly, on the basis of 2 independent SNPs, genetically predicted ALS was associated with an increase in the relative abundance of *Lactobacillales_order* (per 1-unit higher log odds: β, 0.51; 95% CI, 0.09–0.94; *P* = 0.019). Single *Lactobacillales_ORDER*-related SNP effect was estimated and is shown in eFigure 4. The estimated effects of ALS on the microbiota of each genus are listed in eTable 5. No horizontal pleiotropy or outliers were observed.

## Discussion

This study assessed the causal effects of potential microbiome modulators of human ALS and added intriguing evidence implicating some genera of the gut microbiome in modifying susceptibility to ALS. These genera attenuate ALS risk through gamma-glutamyl-related metabolite levels, supporting that a trans-synaptic, glutaminergic, excitotoxic mechanism could provide a pathogenic basis for ALS. These results may contribute to designing microbiome- and microbiome-dependent metabolite interventions in future ALS clinical trials. We further provide genetic evidence that the pathophysiology of ALS is associated with an altered relative abundance of the microbiota, strengthening the bidirectional relationship between the gut microbiota and ALS.

The gut microbiome is a source of these potentially disease-modifying bioactive metabolites and has recently been suggested to contribute to the pathogenesis of neurological disorders [33, 34]. The family *Enterobacteriaceae* includes over 30 genera and 120 species of Enterobacteriaceae, but more than 95% of clinically significant strains fall into 10 genera and fewer than 25 species. All members of the *Enterobacteriaceae* family ferment glucose with acid production and nitrogen metabolism. Glutamine synthetases (GSs) are key enzymes of nitrogen metabolism, and their activity is modulated by nitrogen repression [35]. *Acidaminococcaceae*, an important glutamate-fermenting family of microbes, produces ammonia as the major end product through glutamate fermentation [36]. It is possible that alterations in the microbiomes of the two genera lead to changes in gamma-glutamyl-related metabolite levels. Circulating bioactive gamma-glutamyl-related metabolite levels produced by the gut microbiome permeate the blood–brain barrier, after which they can play important roles in the pathogenesis of brain-related diseases [37].

Our study showed that higher ALS susceptibility was associated with a higher relative abundance of *OTU4607_Sutterella* and *Lactobacillales_ORDER.* In previous studies, gut dysbiosis, particularly reduced levels of butyrate-producing bacteria and higher *E. coli* and *Enterobacteria* abundance, was also found in ALS mice and ALS patients [9, 38]. Furthermore, butyrate and short-chain fatty acids (SCFAs) produced by gut microbiota have been proposed as promising potential therapeutic agents affecting ALS progression [39, 40]. However, unravelling the interplay between the gut microbiome and ALS is imperative, and more direct evidence and results are needed to clarify how the gut microbiota improves or aggravates ALS.

There are several strengths in the present study, including the assessment of genera of gut microbiota and promising metabolites in relation to ALS, the use of data from the largest GWASs to date and bidirectional MR design. This design technique minimizes confounding by known and unknown factors and avoids reverse causation. In addition, consistent results from several sensitivity analyses, including the use of weighted mode, weighted median, and MR-Egger methods, indicate the robustness of our findings. Several limitations merit consideration. First, we used a limited number of gut microbiota and ALS SNPs as IVs; we cannot exclude that our findings might have been affected by weak instrument bias, although all genetic instruments were associated with exposure (*F*-statistic > 10). Second, another potential source of bias in MR analyses is population stratification. We reduced this bias because the dataset for gut microbiota, metabolites and ALS was restricted to individuals of European ancestry. Replication with functionally relevant genetic prediction of gut microbiota is warranted given the substantial difference in gut microbiota composition among different populations. Finally, 16S rRNA gene sequencing only permits resolution from the genus to the phylum level rather than at a more specific level, resulting in biased results if some specific species contributed to ALS.

## Conclusion

Our findings provide novel evidence supporting the bidirectional relationship between the gut microbiota and ALS and highlight that a transsynaptic, glutaminergic, excitotoxic mechanism could provide a pathogenic basis for ALS. These results may contribute to designing microbiome- and microbiome-dependent metabolite interventions in future ALS clinical trials.

## Supplementary Information

**Additional file 1: eTable 1**. Correlation Matrixes for Single Nucleotide Polymorphisms Predicting (a)OTU10032 unclassifed Enterobacteriaceae Species-level OUT and (b)Unclassifed Acidaminococcaceae From SNiPA Pairwise LD.

**Additional file 2: eTable 2.** Associations between gut microbiota and amyotrophic lateral sclerosis in sensitivity analyses.

**Additional file 3: eTable 3.** Associations between gut microbiota and amyotrophic lateral sclerosis in in a leave-one-out approach.

**Additional file 4: eTable 4.** Associations between gut microbiota and amyotrophic lateral sclerosis in sensitivity analyses.

**Additional file 5: eTable 5.** Effect estimates for association of genetically predicted amyotrophic lateral sclerosis with gut microbiota using inverse variance weighting method.

**Additional file 6: eFigure 1.** Association of genetically predicted OTU10032 unclassified Enterobacteriaceae species-level OTU with amyotrophic lateral sclerosis. Squares represent the odd ratios of amyotrophic lateral sclerosisper 1-allele increase in single nucleotide polymorphisms related to greater abundance of OTU10032 unclassified Enterobacteriaceae Species-level OTU; horizontal lines represent 95% confidence intervals (CIs); diamond represent the overall odds ratio with its 95% CI.

**Additional file 7: eFigure 2.** Association of genetically predicted unclassified Acidaminococcaceae with amyotrophic lateral sclerosis. Squares represent the odd ratios of amyotrophic lateral sclerosisper 1-allele increase in single nucleotide polymorphisms related to greater abundance of unclassified Acidaminococcaceae; horizontal lines represent 95% confidence intervals (CIs); diamond represent the overall odds ratio with its 95% CI.

**Additional file 8: eFigure 3.** Association of genetically predicted amyotrophic lateral sclerosis with *OTU4607 Sutterella.* Squares represent the effect estimates of the relative abundance of*OTU4607 Sutterella*per 1-unit higher log odds of amyotrophic lateral sclerosis; horizontal lines represent 95% confidence intervals (CIs); diamond represent the effect size with its 95% CI.

**Additional file 9: eFigure 4.** Association of genetically predicted amyotrophic lateral sclerosis with *Lactobacillalesorder*. Squares represent the effect estimates of the relative abundance of*Lactobacillalesorder*per 1-unit higher log odds of amyotrophic lateral sclerosis; horizontal lines represent 95% confidence intervals (CIs); diamond represent the effect size with its 95% CI.

## Data Availability

Data-set used in the current study is publicly available and not anonymized in this study. The summary datasets analyzed during the current study are available in the http://als.umassmed.edu/#sumstats. The rest datasets used and/or analyzed during the current study are available from the corresponding author on reasonable request.

## Abbreviations

ALS: Amyotrophic lateral sclerosis
MR: Mendelian randomization
AM: *Akkermansia muciniphila*
IVs: Instrumental variables
IVW: Inverse variance-weighted method
SNPs: Single nucleotide polymorphisms
GWASs: Genome-wide association studies
OR: Odds ratio
GS: Glutamine synthetases
