# Supplementary material for: Assessment of bidirectional relationships between 98 genera of the human gut microbiota and amyotrophic lateral sclerosis: a 2-sample Mendelian randomization study
Source: BMC Neurol. 2022 Jan 3;22:8. doi: 10.1186/s12883-021-02522-z (PMC8721912; doi:10.1186/s12883-021-02522-z)
Supplement: Supplementary file 7 — Additional file 7: eFigure 2. Association of genetically predicted unclassified Acidaminococcaceae with amyotrophic lateral sclerosis. Squares represent the odd ratios of amyotrophic lateral sclerosisper 1-allele increase in single nucleotide polymorphisms related to greater abundance of unclassified Acidaminococcaceae; horizontal lines represent 95% confidence intervals (CIs); diamond represent the overall odds ratio with its 95% CI. [file 12883_2021_2522_MOESM7_ESM.pdf]

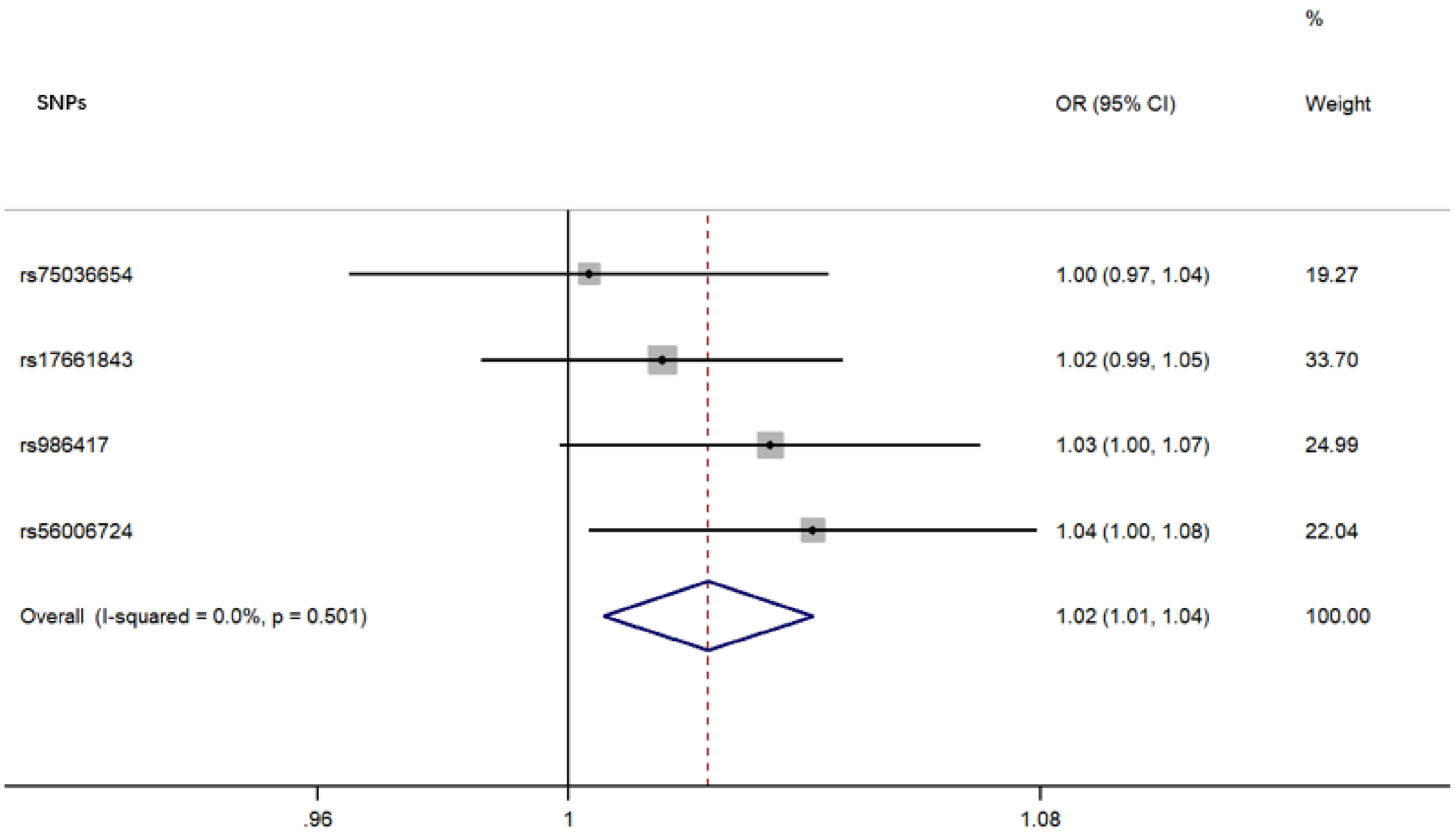

eFigure 2. Association of genetically predicted unclassified Acidaminococcaceae with amyotrophic lateral sclerosis. Squares represent the odd ratios of amyotrophic lateral sclerosis per 1-allele increase in single nucleotide polymorphisms related to greater abundance of unclassified Acidaminococcaceae; horizontal lines represent 95% confidence intervals (CIs); diamond represent the overall odds ratio with its 95% CI.
