# Supplementary material for: Assessment of bidirectional relationships between 98 genera of the human gut microbiota and amyotrophic lateral sclerosis: a 2-sample Mendelian randomization study
Source: BMC Neurol. 2022 Jan 3;22:8. doi: 10.1186/s12883-021-02522-z (PMC8721912; doi:10.1186/s12883-021-02522-z)
Supplement: Supplementary file 8 — Additional file 8: eFigure 3. Association of genetically predicted amyotrophic lateral sclerosis with OTU4607 Sutterella. Squares represent the effect estimates of the relative abundance ofOTU4607 Sutterellaper 1-unit higher log odds of amyotrophic lateral sclerosis; horizontal lines represent 95% confidence intervals (CIs); diamond represent the effect size with its 95% CI. [file 12883_2021_2522_MOESM8_ESM.pdf]

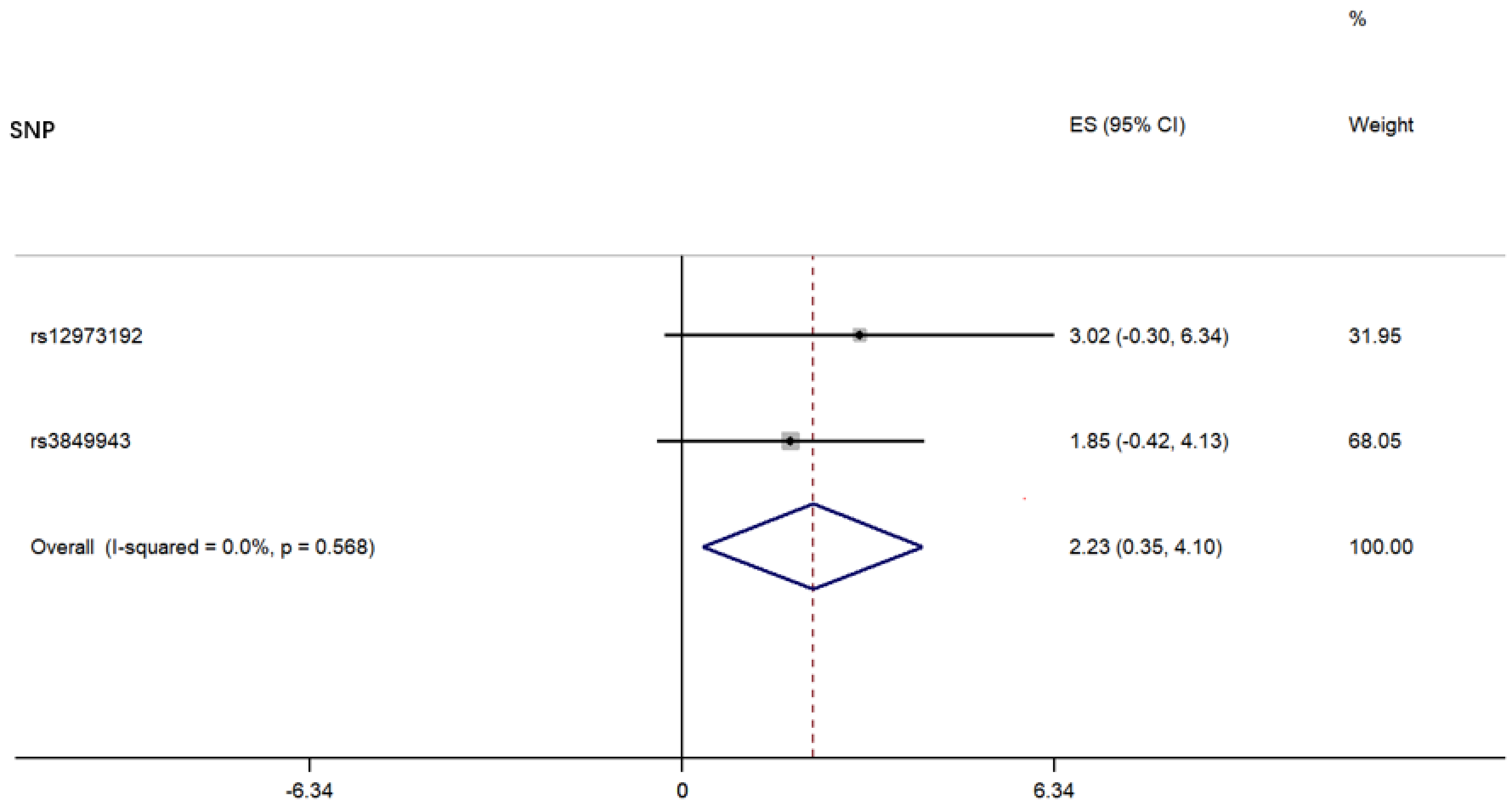

eFigure 3. Association of genetically predicted amyotrophic lateral sclerosis with *OTU4607 Sutterella*.
Squares represent the effect estimates of the relative abundance of *OTU4607 Sutterella* per 1-unit higher log odds of amyotrophic lateral sclerosis; horizontal lines represent 95% confidence intervals (CIs); diamond represent the effect size with its 95% CI.
